# Prognostication and Risk-Adapted Therapy of Hodgkin's Lymphoma Using Positron Emission Tomography

**DOI:** 10.1155/2011/271595

**Published:** 2010-12-22

**Authors:** Yvette L. Kasamon

**Affiliations:** Department of Oncology and Medicine, Johns Hopkins University School of Medicine, 1650 Orleans Street, Baltimore, MD 21231, USA

## Abstract

The use of ^18^F-fluorodeoxyglucose positron emission tomography (FDG-PET) for response assessment in lymphoma is now widespread. Prognostic information obtained from PET performed after two to three cycles of chemotherapy may guide more individualized, risk-adapted therapeutic strategies. Progress in the risk stratification of Hodgkin's lymphoma through midtreatment PET is reviewed, with a focus on management implications in newly diagnosed and relapsed disease. How to tailor treatment on the basis of the interim PET result is not yet defined but is the subject of ongoing trials.

## 1. Introduction

Strategies that minimize toxicity, without compromising disease control, are a leading area of research in Hodgkin's lymphoma (HL). Because the majority of HL patients are cured, investigations can now focus upon reducing treatment toxicities [1, 2]. However, a clear subset of patients has progressive or relapsed disease and may benefit from a preemptive, tailored approach. More accurate prognostication could identify such poor-risk patients, while sparing good-risk patients from the toxicities of overly intensive therapy.

In the assessment of treatment response, there are limitations in the prognostic information provided by computed tomography (CT). Residual masses are frequent after lymphoma therapy, particularly with initial bulky disease [3, 4]. Yet, it is recognized that their presence and size correlate poorly with outcome [3–5]. This underlies the response category of “complete remission unconfirmed” in the 1999 International Working Group criteria [6]. ^18^F-fluorodeoxyglucose positron emission tomography (FDG-PET) enhances the ability to distinguish between viable tumor and fibrosis or necrosis in residual masses [7, 8]. Given this and the increasingly widespread use of PET in response assessment, PET has been integrated into the recently revised response criteria for lymphoma [9, 10]. When performed midtreatment, PET also provides valuable information about the quality of the treatment response and ultimate prognosis in patients with aggressive lymphomas including HL. The prognostication of HL on the basis of early metabolic imaging with PET is reviewed and treatment implications considered.

## 2. Pretreatment Prognostic Indices in HL

The International Prognostic Score (IPS) remains the most commonly used scoring system for newly diagnosed, advanced HL [11]. This consists of seven pretreatment variables that independently predict inferior outcome: albumin <4 g/dL, hemoglobin <10.5 g/dL, male sex, age ≥45 years, stage IV disease, white blood cell count ≥15,000/mm^3^, and lymphocytopenia (lymphocyte count <600/mm^3^ or <8% of the white blood cell count). Each adverse factor is similarly prognostic, reducing the 5-year freedom from progression (FFP) rate by approximately 8%, with the estimated 5-year FFP ranging from 84% with no risk factors to 42% with ≥5 factors in the original paper. The IPS has been utilized in the design and analysis of clinical studies and may guide the choice of chemotherapy in individual patients. 

For early stage disease, cooperative groups have used other baseline criteria for risk stratification and treatment selection [1, 12–14]. Shared factors have included the degree of elevation in erythrocyte sedimentation rate, the number of involved regions, and the presence of bulky disease. Stage IIB is often regarded as advanced disease.

Such pretreatment indices are useful in characterizing groups of patients. However, they are less useful in the prognostication of an individual patient. Furthermore, these are static rather than dynamic parameters and do not provide information about the quality of the treatment response. In contrast, metabolic imaging performed early in the course of therapy provides useful, individualized information about the quality of response and about prognosis.

## 3. Posttreatment PET in HL

For PET scans performed after the end of chemotherapy or chemoradiation for HL, the negative predictive value (NPV, i.e., negative PET scan and no treatment failure) has been consistently high—approximately 90% [15–20]. This is despite some of the studies being restricted to patients with residual masses [15, 18, 20]. In contrast, the reported positive predictive value (PPV, i.e., positive PET scan and treatment failure) is weaker and varies widely, ranging from 26% to 74% in representative series [17–20]. As such, a positive posttreatment PET scan should be interpreted with particular caution, especially within two to three months of RT [9]. 

It must be kept in mind that, even in a reliably FDG avid tumor, a negative PET does not necessarily signify complete eradication of disease [21]. The limit of resolution of current PET systems for detecting tumor generally ranges between 0.5 and 1 cm [22, 23], which translates into an estimated 10^8^ to 10^9^ cells. Therefore, millions of viable tumor cells could persist despite the achievement of a negative PET scan [21]. This has substantial implications for the study of de-escalated therapy in patients with a negative interim PET.

## 4. Midtreatment PET and Prognosis in Newly Diagnosed HL

Earlier identification of chemoresistant disease, prior to treatment completion, would facilitate an individualized, risk-adapted strategy. Midtreatment (interim) metabolic imaging via FDG-PET has strong potential in this regard. In HL and aggressive B-cell non-Hodgkin's lymphoma (NHL), it has been repeatedly recognized that PET performed after only 2 or 3 cycles of chemotherapy provides valuable prognostic information. Early achievement of a negative PET (absence of abnormal FDG uptake concerning for tumor) is prognostically favorable, whereas persistent abnormal FDG uptake on early PET, even in the context of a CT response, raises concern for treatment failure [24]. PET performed after only one cycle also appears to have prognostic significance [25, 26]. 

Why can interim PET provide more valuable prognostic information than one at the end of therapy? An early PET result may offer a window into the chemosensitivity of the tumor. As previously reviewed [21], cancers are usually not diagnosed until they reach a size of 10^10^ to 10^11^ cells. Chemotherapy kills tumor by first-order kinetics; that is, a given dose kills the same fraction, not the same number, of cells regardless of tumor size [27]. Thus, in the idealized setting, each cycle of chemotherapy must kill approximately 1.5 to 2 logs of tumor for a lymphoma to be cured after 6 cycles. Accordingly, most of the tumor cell kill should occur early—with the first 2 cycles [21]. A negative cycle 2 PET implies that the rate of tumor cell kill is sufficient to produce cure by the end of treatment, whereas a negative scan late in treatment does not distinguish between 4 logs of kill (PET remission) and the 10 or 11 logs of kill necessary for cure [21]. 

As in NHL, the literature on the prognostic significance of interim PET in newly diagnosed HL represents a mixture of prospective and retrospective studies (Table 1), utilizing variable scanning techniques and criteria for PET interpretation [25, 28–38]. In a meta-analysis of interim PET in 360 patients with newly diagnosed advanced HL, the sensitivity of interim PET was 0.81 (95% confidence interval, 0.72 to 0.89) and the specificity was 0.97 (95% confidence interval, 0.94 to 0.99); corresponding sensitivity and specificity for diffuse large B-cell lymphoma were 0.78 and 0.87 [24]. 

FDG-PET performed after 2 cycles of ABVD (doxorubicin, bleomycin, vinblastine, dacarbazine) complements and is potentially superior to the IPS [39]. In the widely cited study by Gallamini et al. [31], 260 patients with poorer-risk HL were prospectively evaluated with cycle 2 PET, without change of therapy. The estimated 2-year progression-free survival was 95% in those with a negative interim PET, but only 13% in those with a positive interim PET. On univariate analysis, the cycle 2 PET result and the IPS were among the variables that were statistically significantly associated with treatment outcome. Notably, however, on multivariate analysis, only cycle 2 PET and the presence of stage IV disease were independently associated with outcome. 

The majority of data on the prognostic significance of interim PET in HL is in patients with more advanced disease [31, 32, 36, 38]. Of these, the majority of patients had an IPS of <4 [24]. The prognostic value of interim PET in early stage, favorable disease is not well established. One would expect, given the greater pretest probability of early stage patients being cured, that the high NPV observed in advanced HL would also hold for early stage HL provided that treatment is not de-escalated. In contrast to the high NPV of interim PET, the observed PPV has been variable (Table 1). Furthermore, relatively few patients with positive interim PET have been studied (50 in the largest series) [31]. This is an important consideration when treatment intensification is contemplated on the basis of a positive PET, given the risk of greater toxicity with more intensive treatment.

A number of other factors may account for the observed variability in the PPV of interim PET. As in NHL, the variability is likely due in part to methodological differences among the studies. Variable (but mostly qualitative) criteria have been used in defining a positive scan, with borderline cases of “minimal residual uptake” [30] posing particular challenges as later discussed. There are also differences in image acquisition protocols and technique; for example, earlier studies used PET alone, in contrast to combination PET/CT.

In addition, false positive signals from inflammation are a leading consideration [40], as are other causes of false positive signals including infection, supraclavicular brown fat, granulocyte colony stimulating factors in bone marrow disease [41], bony lesions, and in the case of posttherapy scans, thymic hyperplasia which can mimic mediastinal disease. False positives due to inflammation are possible in any type of tumor, but are a particular consideration in HL given its unique histopathology. In contrast to NHL, less than 1% of the tumor mass in HL is comprised of the malignant Hodgkin and Reed-Sternberg cells; rather, most of the tumor is comprised of nonmalignant cells, with the nature of that cellularity having prognostic significance [42, 43]. This raises the question, what are the histologic underpinnings of a positive PET signal in HL? 

Moreover, the prognostic utility of any criterion can be a moving target, depending on the effectiveness of the underlying regimen. Thus, the prognostic and predictive value of interim PET could vary depending on whether one receives ABVD versus BEACOPP (bleomycin, etoposide, doxorubicin, cyclophosphamide, vincristine, procarbazine, prednisone) [39], or chemotherapy alone versus combined modality therapy. The PPV of interim PET may indeed be lower after BEACOPP [36, 39].

## 5. Risk-Adapted Strategies in Newly Diagnosed HL

Risk-adapted approaches in lymphoma utilizing interim PET are feasible and hold promise [13, 44–46]. Multicenter trials are in progress in patients with newly diagnosed, limited stage [47–49] and advanced stage HL [50–54], evaluating risk-adapted strategies based on the results of interim PET (Table 2). Most of these trials focus upon adult HL, although interim PET is also being investigated in pediatric HL [55].

### 5.1. Risk-Adapted Strategies in Limited Stage HL

In limited stage HL, a central question is whether therapy can be safely de-escalated or abbreviated, including through the omission of RT, in patients achieving a negative interim PET result during initial chemotherapy [47–49]. 

The prognostic information provided by early metabolic imaging may inform decisions about RT in limited stage HL. The role of using interim PET for this purpose cannot be considered standard but is in the process of being defined. Limited stage HL is traditionally treated with combined modality therapy. However, the effectiveness of RT for local control of HL must be balanced by the risks of late toxicities, including second malignancies and cardiovascular disease [56]. Such toxicities are leading causes of morbidity and mortality in patients who are cured of their lymphoma. Concerns about the late effects of RT have led to concerted efforts to define the minimum amount of radiation necessary to maintain cure rates. These include reduction in RT dose (e.g., from 30 to 20 Gray [14]) and the move from extended-field to involved-field [2] to, most recently, involved-node RT [57]. 

The appropriateness of chemotherapy alone for limited stage HL is a matter of debate. Chemotherapy alone has, however, emerged as a viable alternative to chemoradiation, at least in patients with nonbulky disease. A statistically significant overall survival difference has not been conclusively shown with the omission of RT in nonbulky, stage I or II HL [1, 58, 59]. A randomized study that found a difference in favor of RT had methodological limitations [60]. However, the risk of relapse has been higher with the omission of RT [1, 58]. In the HD6 trial of limited stage, nonbulky HL, the 5-year freedom-from progression after ABVD alone was 87%, versus 93% in the group that received RT (*P* = .06) [1]. As such, a minority of patients are affected by the omission of radiation. Of note, the rapidity of the chemotherapeutic response may identify this subset, as the 5-year FFP was significantly better in those who achieved CR or unconfirmed CR by anatomic criteria after 2 cycles of ABVD (95% at 5 years, versus 81% in those who did not; *P* = .007) [1]. It is possible that early prognostication via interim PET may help to differentiate these subsets, thereby identifying who least stands to benefit from the addition of RT. The impact of this approach remains to be determined. In limited-stage HL, three large, multicenter, randomized trials are basing the radiation decision upon the results of PET performed after 2 or 3 cycles of ABVD (Table 2) [47–49]. All three trials investigate the omission of RT in patients who achieve a negative PET after 2 or 3 cycles of ABVD. 

In terms of the number of chemotherapy cycles, caution is advised when considering de-escalating therapy on the basis of a negative interim PET result. The generally favorable outcomes of interim PET negative patients occurred following unabbreviated therapy. In the H10 trial involving PET after 2 ABVD cycles for stage I-II HL, additional chemotherapy is given to the PET negative patients who do not receive RT (Table 2) [49]. Notably, this trial includes patients with either favorable or unfavorable (including bulky) disease. In contrast, in the HD16 trial which is restricted to early favorable disease (stage I-II without risk factors), patients with a negative PET after 2 cycles of ABVD receive no further therapy in the experimental arm [48]. Similarly, in the RAPID trial which is restricted to nonbulky, stage IA or IIA disease, interim PET negative patients who are randomized to the no-radiation arm receive no further chemotherapy [47]. Particularly if RT is omitted, one risks undertreatment if an adequate course of systemic chemotherapy is not delivered, as a negative PET scan may not indicate absence of disease. The results of these trials are awaited. The early cessation of chemotherapy because of a negative interim PET cannot be supported outside of a clinical trial. 

In the risk-adapted management of HL, an additional consideration is that resistance to radiation and resistance to chemotherapy often coexist. This is exemplified by the lower likelihood of radiation eradicating the tumor if relapse or progression occurs shortly (less than a year) after chemotherapy [61]. In addition, tumors that respond suboptimally to chemotherapy commonly relapse or progress within the radiation field. For example, in a retrospective study of 81 HL patients treated with Stanford V chemotherapy, 4 out of 6 patients with a positive PET before pre-planned RT relapsed; 3 of these relapses occurred within the radiation field and one at the margin [62]. RT may also increase the risk of toxicity from subsequent potentially curative therapies such as blood or marrow transplantation (BMT). As such, in patients with positive interim or postchemotherapy PET scans, RT should not necessarily be considered the appropriate next step. Although a true positive PET may identify a subset who stand to benefit from RT, a true positive PET might also identify a subset who stand *not* to benefit from RT.

### 5.2. Risk-Adapted Approaches in Advanced HL

In current risk-adapted trials for advanced HL based on metabolic imaging, the main questions are twofold: (a) whether treatment intensification improves outcome in interim PET positive patients and (b) whether toxicity can be minimized in interim PET negative patients (Table 2).

Improved prognostication could identify which HL patients can benefit the most from intensified but more toxic chemotherapy. Escalated BEACOPP is an effective first-line regimen that, compared to COPP-ABVD (cyclophosphamide-vincristine-procarbazine-prednisone-ABVD) and standard BEACOPP, confers superior FFP and overall survival rates at 5 years [63] and beyond [64] in advanced HL. However, it is also more toxic, including increased risk of acute hematologic toxicity, infertility, and myelodysplasia or leukemia [63]. Although escalated BEACOPP appears to be superior to ABVD in disease control [65, 66], its greater toxicity, combined with the ability to salvage relapsed patients [65], has hindered its widespread acceptance as the new standard of care. 

In the interim PET positive patients, escalation of therapy from ABVD to a BEACOPP regimen is among the approaches under investigation [51, 53, 54]. In the first published study of risk-adapted therapy in HL using interim metabolic imaging [13], 108 patients had therapy tailored using ^67^Ga scintigraphy or PET performed after 2 cycles of standard or escalated BEACOPP. Pretreatment risk factors determined the initial regimen, with that regimen continued, escalated, or de-escalated according to the interim functional imaging result. Treatment failure occurred in 2% of patients with negative interim PET and 27% of patients with positive interim PET. Notably, 79% of the patients initially deemed high-risk had therapy reduced to standard BEACOPP, and overall survival rates were similar in the intermediate-risk and high-risk groups [13]. Although this approach has not been studied in a randomized fashion, the results demonstrate that, even among patients deemed to be high-risk by pretreatment indices, a minority require intensified upfront therapy.

The several ongoing trials involving change from ABVD to BEACOPP in advanced HL [51, 53, 54] will not directly prove the utility of this approach as compared with continued ABVD. They will, however, provide important data that, together with the H10 trial in limited stage HL (which has both standard and experimental arms) [49] will help answer this question. Two trials [50, 53] will also interestingly address the contribution of rituximab, a drug that has activity in classical HL despite the usual absence of CD20 on Reed-Sternberg cells [67, 68].

In the interim PET negative patients, investigational approaches include reduction in the number of chemotherapy cycles, omission of bleomycin (given its risk of pulmonary toxicity), and omission of consolidative RT (Table 2). With respect to the latter, the role of RT in advanced stage HL is undefined [69–72] and practices vary. For example, in a randomized study of advanced stage HL patients achieving an anatomic complete remission to chemotherapy, there was no difference in 5-year survival, and there was a tendency toward inferior survival, with RT [69]. In a meta-analysis of chemotherapy versus chemoradiation for advanced HL, RT was associated with a significantly inferior overall survival [70]. On the other hand, a retrospective study in advanced HL found a progression-free and overall survival advantage to RT, despite more of the radiated patients having initial bulky disease and partial remissions after chemotherapy [72]. BEACOPP, as originally developed, included focal RT to sites of initial bulky disease or residual tumor [63]. More recently, end-of-chemotherapy PET scan has been utilized to limit RT to residually FDG avid masses after BEACOPP, with encouraging preliminary results [15]. The HD18 trial omits RT altogether in patients achieving a negative cycle 2 PET after escalated BEACOPP [50]. Other multicenter trials for advanced HL (HD0801, HD0607) are investigating RT omission in a randomized fashion in patients with negative interim and end-of-chemotherapy PET scans [52, 53].

## 6. Prognostication through Metabolic Imaging in Relapsed or Refractory HL

In patients with HL, high-dose therapy with BMT is reserved for the relapsed or refractory setting. Although an overall survival advantage has yet to be demonstrated, high-dose therapy with BMT prolongs event-free survival in patients with relapsed HL and is potentially curative [73]. In relapsed HL, autologous BMT is associated with a 5-year event-free survival of approximately 40%–50%, although late treatment failures occur [74–76]. 

In both HL and NHL, it is widely recognized that one of the leading determinants of outcome is the sensitivity of the tumor to salvage chemotherapy administered as a bridge to transplantation [74, 75, 77, 78]. Failure rates after BMT are excessive in the context of resistant relapse [79]. CT has been the standard imaging modality for determination of response, and therefore potential chemosensitivity and candidacy for transplantation, following salvage chemotherapy. However, a growing literature suggests that the presence of residual metabolic activity in the tumor prior to transplantation is prognostically significant and may be superior to anatomic response assessment (Table 3) [45, 80–84]. As reviewed previously [85], many of the studies of interim, pretransplantation PET are heterogeneous in terms of disease (combination of HL and NHL, primary refractory and relapsed cases), design (retrospective, prospective), and metabolic response criteria. Table 3 summarizes selected studies of metabolic imaging performed after salvage chemotherapy, prior to high-dose therapy with autologous BMT, in patients with relapsed or refractory HL [45, 81–84]. The literature suggests that residual PET positive disease prior to transplantation portends worse outcomes. This is illustrated by a study by Moskowitz et al. [45], whose group previously found that initial remission under one year, active B symptoms, and extranodal disease were associated with inferior outcomes in relapsed HL. In patients who were chemoresponsive by CT criteria, there was no apparent difference in outcome according to the number of these risk factors provided that pretransplantation metabolic imaging (via gallium scintigraphy or PET) was negative [45].

How might this prognostic information be utilized? Further, prospective studies as to whether metabolic imaging complements pretreatment clinical risk scores in HL would be of interest [82]. Although PET may more accurately identify poor-risk patients who are less likely to benefit from autologous BMT, outcomes are far from all-or-none. The available literature does not support withholding transplantation to an otherwise appropriate, “responding” patient with HL on the basis of a positive interim PET. Whether, however, selected patients stand to benefit more from an alternative salvage approach such as allogeneic transplantation is a question that warrants further study. Rather than relying solely on the chemotherapy or chemoradiation in the preparative regimen, an allogeneic transplant offers the possibility of an immunologic attack against the tumor via a graft-versus-lymphoma effect [74]. This is especially attractive in a lymphoma that responds suboptimally to chemotherapy.

## 7. General Considerations in Analyzing the Midtreatment PET

For the prognostication of HL in clinical trials, performing an interim PET after two or three cycles of chemotherapy seems optimal (Tables 1 and 3). To avoid the transient fluctuations in FDG uptake that may occur after chemotherapy [86] and to permit the chemotherapy to take effect, an interim PET should be performed as close to the next cycle as possible. This consideration is balanced against the logistics of obtaining the centralized read promptly and keeping treatment on schedule, which is feasible [87]. 

In addition to causes of false positive and false negative results, a number of issues surround the interpretation of FDG PET scans that are highly relevant for prognostication. These include the criteria utilized for metabolic response and the reproducibly of the read, as briefly reviewed next.

### 7.1. Definition of Metabolic Response

A baseline PET is advisable to facilitate the evaluation of subsequent scans. Interim or posttherapy PET is most widely interpreted using visual (qualitative) criteria. Yet, FDG uptake lies on a continuum. A challenge in metabolic response assessment is the dichotomization of this continuous variable as either “positive” or “negative” for the purposes of response assessment, prognostication, and therapy. There has been variation among studies (including the ongoing studies of risk-adapted therapy) in what constitutes a “positive” or a “negative” result and the reference background against which tumor FDG uptake is compared (surrounding tissue, mediastinal blood pool, mediastinal blood pool plus normal liver). These criteria have not been validated. Particularly difficult are cases of low-level tumoral FDG uptake, just above background, that are deemed unlikely to represent malignancy but may [29]. The prognostic significance of such “minimal residual uptake” may vary depending on the pretest probability of treatment failure [88]. 

A standardized set of criteria for interim PET analysis is needed [89]. Without this, the results of the risk-adapted studies may be difficult to generalize. The International Harmonization Project criteria utilized for posttreatment response designation were not developed for, nor are recommended for, midtreatment response assessment [9]. These criteria may be prone to high rates of interim PET positivity; any activity above local background in a less than 2 cm mass including in normal size lymph nodes is considered positive (to account for effects of partial volume averaging), as is any activity above mediastinal blood pool in a larger mass [9]. A prospective trial is evaluating a cutoff of 1.5 times blood pool activity for differentiating between positive and negative results in NHL [90].

In addition to whether a PET is “positive,” the intensity of residual FDG uptake may have prognostic significance [44]. A 5-point scale (the Deauville criteria [91], based on the “London” criteria [88]) that captures gradations in FDG uptake has been proposed for interim response assessment, with scores of 4 or 5 considered “positive”:

1. no uptake above background,

(2) uptake ≤ mediastinum,

(3) uptake > mediastinum but ≤ normal liver,

(4) uptake moderately > liver,

(5) uptake markedly > liver and/or new disease.

International validation studies of the 5-point scale are in progress. As with any criteria that categorize a continuous variable, discriminating subtle differences between FDG uptake in tumor as compared to mediastinum or liver may be challenging. Differences in outcome in patients with a score of 2 versus 3 will be of particular interest in studies of interim PET. For the purposes of prognostication and clinical trial planning, what should define a positive or a negative interim PET result? This is not yet known, but may depend upon the risks of the planned intervention. For instance, if intensification (with risks of overtreatment and greater toxicity) is considered on the basis of a positive interim PET, a conservative approach to PET interpretation may be prudent, with borderline cases scored as negative [44]. If de-escalation is considered on the basis of a negative PET, it may be prudent to score such cases as positive so as to avoid undertreatment. 

Semiquantitative assessments using standardized uptake values (SUVs) may complement visual criteria, at least in NHL [92, 93], but have not been shown to be superior to qualitative assessments. This clearly warrants further study, however, as PET is inherently a quantitative imaging method [94]. Criteria for response assessment in solid tumors based on SUVs have been proposed [94].

### 7.2. Reproducibility of the PET Read

Discordance in the PET interpretation is a recognized concern, even if performed by expert nuclear medicine physicians. The interobserver variability is exemplified by an expert panel's analysis of interim PET scans in diffuse large B-cell lymphoma, wherein there was only moderate agreement using predefined visual criteria including the London criteria [95]. Additionally, no consensus could be reached in 9 of 12 discordant cases, with sites of para-aortic disease, spleen, and bone posing particular difficulty [95]. In a multicenter trial for stage II–IV HL, concordance in reads among four centers has been preliminarily studied using the London criteria [88]. Very good agreement (kappa statistic, 0.85) was found for a conservative reading (“positive” defined as a score of 4 or 5), and good agreement (kappa statistic, 0.79) for a sensitive reading (“positive” defined as a score of 3, 4, or 5) [88]. Other investigations of the reproducibility of PET reads in multicenter trials are in progress. Given the potential for greater interobserver reproducibility of semiquantitative measures [94, 96], further efforts to develop and validate semiquantitative criteria are encouraged. The reproducibility of the PET interpretation is key if treatment decisions are rendered on the basis of the PET result. Coupled with the need for standardized, reproducible reporting is the clear need for quality-control measures and consistent scanning techniques in multicenter trials [97].

## 8. Conclusions

In HL, as in NHL, early prognostication through interim PET clearly has the potential to guide optimal treatment. The metabolic imaging result has changed the definitions of disease response and chemosensitivity. However, many methodological and management questions remain. Although it is tempting to incorporate interim PET scanning into routine practice [98], such imaging is best performed on a clinical trial. How to manage the result is not yet established, but will be clarified through ongoing and planned clinical trials.

## Figures and Tables

**Table 1:** PET during first-line chemotherapy for Hodgkin's lymphoma.

Study	Prospective	No.	Stage	Chemo, ±RT	Cycles before PET	No. PET+ (%)	PPV	NPV	EFS, PET+	EFS, PET–	Median follow-up
Friedberg et al. 2004 [28]	yes	22	I–IV; 28% III-IV	Mostly ABVD	3	5 (23%)	80%	94%	—	—	24 mo

Hutchings et al. 2005 [29]	no	85	I–IV; 33% III-IV	Mostly ABVD	2-3	13 (15%)^a^	61.5%	94%	46% (2 y)	97% (2 y)	40 mo
									39% (5 y)	92% (5 y)	

Gallamini et al. 2006 [34]	yes	108	IIA with RF, IIB-IV; 46% III-IV	Mostly ABVD	2	20 (19%)	90%	97%	6% (2 y)	96% (2 y)	20 mo (mean)

Hutchings et al. 2006 [30]	yes	77	I-IV; 36% III-IV	Mostly ABVD	2	16 (21%)^a^	69%	95%	0% (2 y)	96% (2 y)	23 mo
		64			4	13 (20%)	85%	96%	19% (2 y)	96% (2 y)	23 mo

Zinzani et al. 2006 [32]	yes	40	IIB-IV; 48% III-IV	ABVD	2	8 (20%)^b^	100%	100%	—	—	18 mo
		40			4	7 (18%)^b^	100%	100%	—	—	18 mo

Kostakoglu et al. 2006 [25]	no	23	II–IV; 35% III-IV	ABVD	1	6 (26%)	83%	100%	17% (2 y)	100% (2 y)	20 mo

Querellou et al. 2006 [33]	no	44	IIA-IV; 63% III-IV	Mostly ABVD	3-4	—^c^	—	95%^c^	—	95% (1 y)	18 mo

Gallamini et al. 2007 [31]	yes	260^d^	IIA with RF, IIB-IV; 47% III–IV	Mostly ABVD	2	50 (19%)^a^	86%	95%	13% (2 y)	95% (2 y)	26 mo

Sher et al. 2009 [35]	no	46	Mostly I-II	ABVD-based^e^	—	20 (43%)	15%	96%	85% (2 y)	96% (2 y)^f^	—

Markova et al. 2009 [36]	no	50	IIB with RF, III–IV; 78% III–IV	BEACOPP	4	14 (28%)	14%^g^	97%^h^	86% (2 y)^g^	97% (2 y)	25 mo

Furth et al. 2009 [37]	yes	40	I–IV; 48% III-IV	OEPA^i^	2	14 (35%)	14%	100%	86% (4 y)	100% (4 y)	46 mo

Avigdor et al. 2009 [38]	yes	45	IIB-IV; 93% III–IV	BEACOPPesc × 2, then ABVD^j^	2	13 (29%)	45%	87%	53% (4 y)	87% (4 y)	48 mo

ABVD: doxorubicin, bleomycin, vinblastine, dacarbazine; BEACOPP: bleomycin, etoposide, doxorubicin, cyclophosphamide, vincristine, procarbazine, prednisone; EFS: event-free survival; esc: escalated; MRU: minimal residual uptake; NPV: negative predictive value; OEPA: vincristine, etoposide, prednisone, doxorubicin; PPV: positive predictive value; RF: risk factor(s); RT: radiation therapy. Definition of an event variably includes relapse or progression, incomplete remission, disease-related death, and/or any death; starting points for EFS estimates vary. Table modified from Kasamon et al. [21].

^a^MRU cases were analyzed with PET− cases.

^b^MRU cases (4 after cycle 2 PET, 3 after cycle 4 PET) were analyzed separately; 1 relapsed.

^c^4/24 patients were PET+, 1 being newly diagnosed; 18/20 PET− patients were newly diagnosed.

^d^Includes previously reported patients [30, 34].

^e^All received radiation.

^f^For interim and post-chemotherapy PET− disease; 1/26 was interim PET−, post-chemotherapy PET+.

^g^7/14 received RT, which was restricted to >2.5 cm, PET+ masses on post-chemotherapy imaging.

^h^Includes 1 non-relapse death.

^i^Pediatric study; 98% received RT.

^j^Response-adapted study; with or without additional therapy.

**Table 2:** Current risk-adapted studies using interim PET in Hodgkin's lymphoma.

Study	Group	Projected accrual	Timing of PET	Treatment
Limited disease:				
RAPID trial [47]	UK NCRI	1600	After ABVD × 3	PET−, randomize to RT versus no further therapy
				PET+, further ABVD + RT
HD16 [48]	GHSG	1100	After ABVD × 2	Standard arm: RT regardless of PET
				Experimental: PET−, no further therapy
				Experimental: PET+, RT
H10 [49]	EORTC, GELA, IIL	1600	After ABVD × 2	Standard arm: complete ABVD + RT regardless of PET
				Experimental: PET−, complete ABVD (no RT)
				Experimental: PET+, BEACOPPesc then RT

Advanced disease:				
HD18 [50]	GHSG	1500	After BEACOPPesc × 2	PET−, randomize to 2 versus 6 more cycles (no RT)
				PET+, randomize to BEACOPPesc with versus without rituximab^a^
HD0607 [53]	GITIL	450	After ABVD × 2	PET−, complete ABVD; if still PET–, randomize to RT versus no RT
				PET+, randomize to BEACOPPesc with versus without rituximab^b^
RATHL [51]	UK NCRI	1200	After ABVD × 2	PET−, randomize to ABVD versus AVD (no RT)
				PET+, BEACOPP-14 or BEACOPPesc
HD0801 [52]	IIL	300	After ABVD × 2	PET−, complete ABVD; if still PET–, randomize to RT versus no RT
				PET+, high-dose therapy with autologous BMT
S0816 [54]	SWOG intergroup	230	After ABVD × 2	PET−, further ABVD
				PET+, BEACOPPesc^c^

ABVD: doxorubicin, bleomycin, vinblastine, dacarbazine; BEACOPP: bleomycin, etoposide, doxorubicin, cyclophosphamide, vincristine, procarbazine, prednisone; BMT: blood or marrow transplantation; EORTC: European Organization for Research and Treatment of Cancer; esc, escalated; GELA: Groupe d'Étude des Lymphomes de l'Adulte; GHSG: German Hodgkin Study Group; GITIL: Gruppo Italiano Terapie Innovative Nei Linfomi; IIL: Intergruppo Italiano Linfomi; NCRI: National Cancer Research Institute; RATHL: response-adapted therapy in Hodgkin lymphoma; RT: radiation therapy; SWOG: Southwest Oncology Group.

^a^RT restricted to residual ≥2.5 cm, PET+ sites on end-of-chemotherapy imaging.

^b^If PET− after 4 cycles of BEACOPPesc ± rituximab, changed to standard BEACOPP ± rituximab.

^c^Standard BEACOPP if human immunodeficiency virus positive.

**Table 3:** Selected studies of pretransplantation PET in relapsed/refractory Hodgkin's lymphoma.

Study	Prospective	Type of FI	No.	No. FI+ (%)	PPV	NPV	EFS, FI+	EFS, FI–	Median follow-up
Spaepen et al. 2003 [81]	no	PET	19	9 (47%)	78%	90%	—	—	—

Schot et al. 2007 [82]	yes	PET	23	5 (22%) NMR, 10 (43%) PMR	60% if NMR, 70% if PMR	75% if CMR, 30% if PMR	40% (2 y) if NMR, 37% if PMR	73% (2 y) if CMR	—

Jabbour et al. 2007 [83]	no	^67^Ga or PET	211	57 (27%)	74%	68%	23% (3 y)	69% (3 y)	34 mo for nonrelapsed pts
		PET	68	25 (37%)	72%	77%	—	—	—

Castagna et al. 2009 [84]	no	PET, after 2 cycles	24	10 (42%)	90%	93%	10% (2 y)	93% (2 y)	24 mo
		PET, after 4 cycles	24	6 (25%)	—	—	0% (2 y)	78% (2 y)	24 mo

Moskowitz et al. 2010 [45]	yes	^67^Ga or PET	105	41 (39%)	—	—	33% (4 y)	77% (4 y)	7 y in surviving pts

CMR: complete metabolic remission; EFS: event-free survival; FI: functional imaging; Ga: Gallium; NMR: no metabolic remission; NPV: negative predictive value; PMR: partial metabolic remission (residual intensity above background level); PPV: positive predictive value; pts, patients.
